# Does Really One in Ten Believe Capital Punishment Exists in a Contemporary European Community Country? An Endorsed, Prereviewed, Preregistered Replication Study and Meta-Analysis

**DOI:** 10.3389/fpsyg.2019.01601

**Published:** 2019-07-19

**Authors:** Magdalena Boch, Ulrich S. Tran, Martin Voracek

**Affiliations:** Department of Basic Psychological Research and Research Methods, Faculty of Psychology, University of Vienna, Vienna, Austria

**Keywords:** cultivation theory, death penalty, preregistration, close replication, conceptual replication, meta-analysis, small-telescopes analysis, Mokken scale

## Abstract

**Background:**

Till et al. (2016) reported that in an Austrian sample approximately one in ten respondents incorrectly believed that Austria still practices, or recently practiced, the death penalty, and that there is a positive association between the amount of weekly television viewing and this gross misperception of the Austrian justice system.

**Methods:**

An endorsed, prereviewed, preregistered close (*N* = 597) served to test the veracity of these reported effects. This was coupled with the conceptual extension part, which (a) investigated the potential influence of watching American crime series, (b) accounted for further possible confounds, and (c) tested the generalizability of the effect of television viewing to online streaming.

**Results:**

Online survey data (*N* = 597) replicated the one-in-ten prevalence of incorrect answers with the 5-item death penalty questionnaire used in the original study, but not, when asking directly about Austria’s death penalty practices (prevalence: 0.3%). Younger age, but not the amount of television viewing or online streaming, suggestibility, or preferred TV genre consistently predicted incorrect answers in the death penalty questionnaire. Incorrect answers were Mokken-scalable (i.e., formed a common scale, complying with a non-parametric item response model) and were highly consistent. In contrast to the replication study results, a small meta-analysis of all available evidence (three studies, including the present replication) suggested that the aggregate effect of television viewing nominally was significant, albeit small.

**Conclusion:**

The replication study yielded mixed results, which indicate the perception of a high prevalence of beliefs that there is capital punishment in a country without death penalty probably is due to a faultily designed questionnaire and thus a research artifact. Also, positive associations of television viewing with such beliefs likely are only small at best.

## Introduction

Can television (TV) influence the perception of the real world? According to cultivation theory, the answer is yes (Gerbner et al., 1980, 1986). In this framework, television is described as a socializer, noticeably influencing individuals’ conceptions of reality. The more time is spent watching television, the less time is left for exploring the real world. Consequently, individuals watching a lot of TV might believe that, for example, assumptions or stereotypes seen on TV also apply to the real world and, in turn, might utilize such erroneous information to form biased judgments. Hence, TV may bring about source confusion, i.e., heavy TV viewers may confuse fact for fiction and fiction for fact (Mares, 1996). In line with cultivation theory, Till et al. (2016) summarized previous studies in the field of media psychology, citing evidence for biased judgments about real-world events as a result of TV consumption. However, they noted that research so far has only focused on *biased* judgments and expectations about the real world (so-called first-order effects) and the resulting behavior (so-called second-order effects) of television exposure. For example, TV watching might bias the expected probability to be a crime victim (a first-order effect) and may lead to an increased fear of walking the streets alone at night (a second-order effect; Till et al., 2016). In contrast to previous research, Till et al. (2016) went one step further and argued that TV consumption may not only distort expected probabilities and perception, but might as well lead to *entirely false beliefs*.

In their study, Till et al. (2016) queried individuals from the Austrian general population regarding their beliefs about the death penalty in Austria. As in all other European Community nations, death penalty has been abolished in Austria quite a time ago (formally 1958 in civil law and 1968 in military law, although the last execution was back in 1950; Morscher, 2007). Like in many other Western industralized countries, Austrian television features a considerable volume of American television programs (Bouchehri, 2010 as cited in Till et al., 2016), which in turn consists of a large volume of American crime thrillers and detective series (Gerbner et al., 1980, 1986). Because death penalty in the United States of America still is in effect, Till et al. (2016, p. 540) hypothesized that “the more television Austrians watch, the more likely they are to inaccurately assume that capital punishment exists within Austria.”

Indeed, Till et al. (2016) found a positive correlation between weekly hours spent watching TV and at least one false belief about Austria’s current or recent practice of the death penalty in a 5-item questionnaire. This questionnaire queried participants about the number of current inmates on death row in Austria, and on the numbers of inmates executed with the electric chair or lethal injection in the past 5 and 25 years, respectively (see Materials and Methods). In the absence of death penalty in Austria, any stated number greater than zero on any of these items indicated the presence of a false belief. The effect of TV consumption also proved to be robust when controlling for sociodemographic variables. The study rationale and the 5-item questionnaire were taken from an unpublished thesis of one of the paper’s co-authors (Truong, 2011), who had previously conducted a similar survey with Canadian university students, wherein the effect of TV viewing did not reach nominal significance, however.

### Endorsed, Prereviewed, and Preregistered Replication

The findings of the original study (Till et al., 2016) indicate that approximately one tenth of their sample erroneously believed that death penalty still exists, or recently existed, in Austria. Since this is a fundamental mismatch, as compared with the actual criminal law in Austria, this surprising finding was widely covered in the media (e.g., derStandard, 2016; Kronen Zeitung, 2016; ORF Science, 2016; Vienna Online, 2016). The goal of the present study was to help create a stronger foundation for future research along these lines, because, overall, the results of the original study would be of high practical applicability and theoretical importance with regards to the suggested influence of television on misperceptions of state laws.

Regarding the recent discussion surrounding the replicability of research findings in psychology and other empirical research fields (e.g., Ioannidis, 2005; Open Science Collaboration, 2015), we conducted a preregistered replication study to test the robustness and replicability of the reported effects of Till et al. (2016). Furthermore, the non-significant results of Truong (2011), being still unpublished, might be seen as typical for the prevailing publication bias in psychology and elsewhere (e.g., Rosenthal, 1979; Dwan et al., 2008; Fanelli, 2012; Masicampo and Lalande, 2012; Lakens, 2015), a problem which we aimed to counteract by preregistering our replication attempt at the Open Science Framework (OSF).

Our replication study is comprised of three parts: a close replication of the original study (Till et al., 2016), a conceptual extension to this, and a meta-analytic evaluation of the original findings alongside the replication findings. The close replication part investigated the replicability of the original findings and was designed to match the original study by Till et al. (2016) as closely as possible. Using the original 5-item questionnaire, the replication reassessed the two main findings of the original study, i.e., the prevalence of the erroneous belief that Austria still or recently practiced the death penalty (held by 11.6% of survey respondents, 95% *CI* [7.07%,16.13%]), and the positive relationship of such erroneous beliefs with respondents’ weekly TV consumption (*r* = 0.19, *p* < 0.01, 95% *CI* [0.10,−0.29]). The focus of the data analysis for close replication part was on the associations of TV consumption with the dichotomized score of the sum of all five items of the death penalty questionnaire, rather than with the scores of its individual items (exactly following Till et al., 2016). Based on the findings reported in the original study, the aggregate of the 5-item questionnaire appears to be highly internally consistent (Cronbach α = 0.89; calculated by us on the basis of data reported in Till et al., 2016); examining individual items separately thus appeared to be less informative. However, results with regards to the individual items are presented in this study as well.

The conceptual extension part took a closer look at the 5-item death penalty questionnaire and compared the results therefrom to those from a direct question on whether or not Austria still has the death penalty. Furthermore, the extension part explored the additional hypothesis that asking participants repeatedly about the numbers of inmates on death row, or who had been executed, with an open-response format might lead respondents with higher trait suggestibility to (falsely) assume that zero might not be a correct response option for such questions. Thus, suggestibility and its possible associations with false beliefs on the 5-item death penalty questionnaire were assessed as well.

Moreover, to be able to draw valid conclusions from the death penalty questionnaire, the probability to answer any item correctly should solely derive from participants’ level of beliefs and not from other systematic influences; in other words, items should be stochastically independent (van Schuur, 2011). In the questionnaire, the time range asked for by one item included the full time range asked for by another item (“last 25 years” vs. “last 5 years”). For this reason, it seemed at the outset questionable that such items should be stochastically independent. Hence, we sought to elucidate respondents’ answer patterns on this set of items by applying Mokken scale analysis (van Schuur, 2003; a non-parametric item-response theory equivalent of the Rasch model; Rasch, 1960).

With regards to TV viewing, Till et al. (2016) asserted that Austrians mainly watch American crime and detective series. This claim was based on evidence from previous studies, but not on specific TV viewing habits in their own sample (i.e., preferred genres were not assessed). Therefore, we tested this assumption more directly. Moreover, should the media consumption effect of law misperceptions be veridical, a similar association as with TV consumption should occur with online streaming (watching TV series and films online) as well. Nowadays, individuals might even use streaming services more often than traditional (i.e., “offline”) TV viewing.

Finally, Till et al. (2016) focused on education and age as sociodemographic moderator variables for the effect. In the extension part of our study, length of stay in Austria and nationality were additionally examined. Thus, the purpose of the conceptual extension part was to account for possible additional confounds, in order to address possible methodological issues and to extend the original findings.

The final part of the present study evaluated the replication finding by using small-telescopes analysis (Simonsohn, 2015) and further provided a more robust estimate of the size of the effect under scrutiny, by meta-analytically combining the results from Till et al. (2016) and Truong (2011), and the present close replication, thus following the recommended approach and philosophy of continuously cumulating meta-analyses (CCMA; Braver et al., 2014).

### Replication Endorsement and Preregistration

After the initial preparation of the replication plan, the first author of the original study (Benedikt Till) study was informed about the replication attempt. In turn, he provided consultation and access to the original study materials, as well as to the unpublished thesis of Truong (2011). The replication plan, including methods, hypotheses, analysis plan, and study materials, as well as a first draft of the introduction section were preregistered on the OSF (Boch and Voracek, 2017, June 12) prior to data collection.

## Materials and Methods

### Sample

An *a priori* safeguard power analysis (Perugini et al., 2014), based on the lower boundary of the 80% confidence interval of the original study’s effect size (*r* = 0.19; Till et al., 2016, p. 541), was carried out, to prevent overestimating the target effect size. The power analysis suggested that a study with a sample size of *N* = 293 would be sufficiently powered to detect the target effect of the original study. However, as the original study already had a larger sample size (*N* = 322), the target sample size for the replication study was set to *N* ≥ 322, with a stopping rule of *N* = 400, in order to have more, or at least equal, power as the original study had.

In contrast to Till et al. (2016) – but endorsed by the original author – the present study was conducted online, instead of using a classic paper-and-pencil format. The target population for study participation remained the same, namely the Austrian general population, and data collection proceeded via forwarding invitations to undergraduate students and by using social media platforms. As in the original study, participation was voluntary and without any remuneration. The first page of the survey contained a short study description and information for study participants. Participants provided their informed consent to partake in the survey via clicking a button which also was required to start the online survey. Participation in this online study was anonymous from the outset; there was no written informed consent form. Participation neither affected the physical or psychological integrity, the right for privacy, nor other personal rights or interests of participants. Such being the case, this study was therefore exempt from formal ethical approval, according to national laws (Austrian Universities Act 2002).

Data collection started on June 13, 2017 and lasted until June 16, 2017. Upon reaching a sample size of *N* = 400, the online survey was set to close by the end of the same day. In total, *N* = 623 persons accessed the survey.

In consultation with the original author, the *a priori* criterion to exclude participants who reported an unrealistic amount of weekly TV viewing or streaming was set to 170 h per week (i.e., more than 24 h a day), which however, resulted in the exclusion of a single participant. In addition, a screening question at the beginning of the questionnaire was included to check if all participants currently lived in Austria at the time of responding to the survey. Individuals responding to this question with “No” (*n* = 25) were thanked and dismissed; all others were forwarded to complete the survey.

The final sample for analysis comprised of *N* = 597 respondents (70.5% women; *M*_age_ = 32.85, *SD* = 11.04 years, ranging from 15 to 83 year), who at the time of the survey lived in Austria. The majority of the sample (86.6%) also reported holding the Austrian citizenship. Regarding educational levels, 2.8% (*N* = 17) had completed compulsory education, 9.2% (*N* = 55) apprenticeship training, 5.7% (*N* = 34) intermediate technical and educational school, 32.2% (*N* = 192) had graduated from high school or a secondary school, and 50.1% (*N* = 299) reported a college or university degree as their highest completed school level. As compared to Till et al. (2016), the replication sample had slightly fewer male participants (39% vs. 46%) and was more educated. The original study already had lower educational levels underrepresented, as compared to the general Austrian population (19% compulsory education, 34.3% apprenticeship training, 15.1% intermediate technical and educational school, 17.4% high school or secondary school, 14% college or university; Statistik Austria, 2015).

Additional monitoring of online media coverage surrounding topics about capital punishment during data collection revealed that at the time of, and shortly before, data collection, the topic of death penalty did not appear prominently (i.e., in major headlines) of national or international media outlets.

### Close Replication Measures

#### TV Viewing Frequency

As in Till et al. (2016), weekly TV consumption was assessed using two questions with an open-response format. Respondents were asked to state the average amount of hours spent watching TV on a typical weekday (Monday to Thursday) and on a typical weekend day (Friday to Sunday).

#### Death Penalty Questionnaire

The 5-item death penalty questionnaire to assess participants’ beliefs about current and recent practices of death penalty in Austria was taken from Till et al. (2016, p. 540), but with the year date updated accordingly. Respondents answered to the following questions (in German): (a) How many inmates in Austria do you think are currently sitting on death row (i.e., the number of inmates awaiting execution)? (b) How many inmates in Austria do you think were executed by lethal injection over the past 5 years (2012 to 2016)? (c) How many inmates in Austria do you think were executed by lethal injection over the past 25 years (1992 to 2016)? (d) How many inmates in Austria do you think were executed by electric chair over the past 5 years (2012 to 2016)? (e) How many inmates in Austria do you think were executed by electric chair over the past 25 years (1992 to 2016)?

Each of these questions was accompanied with the instruction, “Please write down the respective number, e.g., 6.” The numerical answers were dichotomized, thus either indicating the answer was correct (zero) or incorrect (any integer larger than zero). Consequently, the questionnaire’s sum score was coded as correct, if participants responsed to all five items with the number zero. Internal scale consistency (Cronbach α) for the questionnaire in Till et al. (2016) was 0.89 (our calculation, based on statistics reported in that paper). In line with the original study from Till et al. (2016), both the individual items and their sum score were used as dependent variables in the replication study (but not simultaneously in the same model).

### Conceptual Extension Measures

#### Length of Stay in Austria

The amount of time participants had lived in Austria was assessed with one question with an open-response format asking how long respondents had been living in Austria. All answers were transformed into years. When ranges were stated, the mid-range (i.e., midpoint of the range) was taken for analysis.

#### Online Streaming Frequency

The weekly number of hours spent using online streaming services was assessed with two questions with open-response format. Respondents stated the average number of hours spent using online streaming services on a typical weekday (Monday to Thursday) and on a typical weekend day (Friday to Sunday).

#### TV Genre Preferences

To examine whether American crime and detective series were part of the most frequently watched TV genres, respondents were asked to select the top-three genres they watched from a list consisting of eleven categories (adapted from Statista, 2017). Respondents could choose between action (e.g., superheroes, adventure, western), crime/detective international, crime/detective national and European, science fiction/fantasy, soap/telenovela, mystery/horror, hospital/doctor, animation, “heimat” (i.e., films with a regional background)/family, comedy (e.g., sitcom, satire), drama (e.g., thriller, politics), and history. The latter two categories were mistakenly not listed in the preregistration of our replication, but all genres were included in the preregistered German translation of the survey form used in the replication study. To prevent sequence effects, genre items were presented in randomized order. If crime/detective international was included in the top-three rankings, these respondents were coded as regular consumers of American crime and detective series.

#### Suggestibility

Trait suggestibility was assessed with the German version of the Short Suggestibility Scale (SSS). This 21-item self-report measure is a short form of the 95-item Multidimensional Iowa Suggestibility Scale (MISS; Kotov et al., 2004). Kotov et al. (2004) report that the SSS strongly correlates with MISS total suggestibility scores (*r* = 0.93) and is internally consistent (Cronbach α > 0.80).

Respondents stated to what extent each statement (examples: “A good salesperson can really make me want their product”; “I frequently change my opinion after talking with others”) applied to them on 5-point Likert scales, ranging from 1 (*not at all*) to 5 (*a lot*). The sum score of all 21 items served as indicator for suggestibility, with higher scores representing higher suggestibility levels.

#### Direct Question on Death Penalty in Austria

To examine whether respondents thought that capital punishment is currently part of the Austrian justice system, a direct question at the end of the survey asked: “Does Austria currently practice the death penalty?”, with “No” being the correct answer. Notably, the respondents were not able to go back to the 5-item questionnaire.

### Procedure

Benedikt Till provided the original study materials, procedural details, and further information on the study’s background, which were adopted, or accounted for, in the close replication part. A detailed description of this part, along with the German-language survey is retrievable on the OSF project site (Boch and Voracek, 2017, June 13). The original study and the close replication study only differed in that the replication study was conducted online and that one additional sociodemographic variable was assessed (asking how long respondents were living in Austria). The original study author did not express any concerns about these changes. In the online survey, the conceptual extension part followed the close replication part. To further prevent any confounding effects, the online-survey respondents were not able to turn back pages.

### Confirmatory Analysis

The following analysis plan was preregistered prior to data collection on the OSF project site (Boch and Voracek, 2017, June 12). Data analyses deviating from the original analysis plan are explicitly marked as such.

#### Close Replication Part

First, we calculated the same intercorrelation matrix of study variables as in Till et al. (2016), in order to compare the replication results with the findings of the original study.

As in Till et al. (2016), six separate binary logistic regression analysis models were calculated, with the death penalty questionnaire’s five individual items, or the sum score of these items combined as the outcomes. In these models, the weekly amount of TV viewing served as a predictor; further, age and education were included as control variables. In line with the original study, the forced-entry method of predictors was applied for all regression models, and continuous variables were standardized prior to model entry. The original study did not account for multiple hypothesis testing; we report both Bonferroni-corrected and uncorrected *p-*values.

#### Conceptual Extension Part

For the conceptual extension part, the relationship between TV viewing and the item directly asking about death penalty was investigated by correlating the online streaming variable with the death penalty questionnaire’s sum score and with the direct question. A paired-group *t*-test was calculated to compare the stated weekly hours of watching TV with those of online streaming.

Cronbach α was calculated for the 5-item death penalty questionnaire as a measure for its internal consistency. In addition, to compare stated prevalences (original study vs. our replication) of inaccurate assumptions on capital punishment in Austria, 95% *CI*s for the proportion of incorrect responses were calculated for the questionnaire’s dichotomized total score, as well for the direct question about death penalty. Diverging from the preregistered analysis plan, 95% *CI*s for the prevalence of incorrect answers on the direct question were bootstrapped, based on 1000 samples. Due to the low point estimate obtained on this question, a symmetric *CI* would have resulted in a negative value for the lower *CI* limit. Furthermore, the association between the dichotomized total score and the direct question was quantified via their odds ratio (*OR*).

In terms of preferred TV genre, percentages and frequencies for each TV genre ranked as top three were calculated. To examine the influence of largely watching American crime and detective series, *OR*s were calculated between the dichotomized total score of the 5-item questionnaire and preference for the respective TV genre. Logistic regression models were utilized to predict the overall performance in the death penalty questionnaire, based on weekly TV viewing times. This analysis accounted for respondents’ age, education, most-watched TV genre (international crime vs. all other), the time participants spent living in Austria, and suggestibility scores. Regression model diagnostics (variance inflation factors) did not indicate any case of multicollinearity, specifically not with regards to the variables age and duration of living in Austria.

Regarding the 5-item death penalty questionnaire, Mokken scale analysis (van Schuur, 2003) was applied to test whether psychometrically the items were part of one meaningful scale, and to quantify the prevalence of illogical response patterns (psychometrically, these are violations of item transitivity; as explained below). Stochastic independence of items was assessed by calculating homogeneity coefficients (*H*_*ij*_) for each item pair and for each item (*H_*i*_)* separately. The homogeneity coefficient for the entire scale (*H*) provided information on the monotonicity of the total scale score. *H* > 0.30 is the recommended cut-off to include an item into a Mokken scale and to consider the whole scale as sufficiently consistent. H > 0.50 indicates a strong Mokken scale. Finally, the number of transitivity violations was assessed. Transitivity refers to the following assumption in the Mokken model: if a respondent correctly answers a more difficult question B, than an easier question A should have been answered correctly as well (van Schuur, 2003). This logic is straightforwardly applicable to the 5-item death penalty questionnaire: any respondent who affirms the question that executions took place within the past 5 years in Austria, must also affirm the question that executions took place within the past 25 years in Austria (because the past 5 years are a subset of the past 25 years). Not doing so would be illogical, and would constitute a case of violated item transitivity. The Mokken scale analysis was done using the R package mokken (van der Ark, 2007, 2012).

#### Cumulative Evidence, Evaluation, and Detectability

To estimate the relationship between the amount of TV viewing and at least one incorrect item response in the death penalty questionnaire (based on the dichotomized sum score), a CCMA (Braver et al., 2014) was conducted, combining the effects from Truong (2011), namely, *r* = 0.14 (*p* = 0.06; calculated by us, based on the reported data), Till et al. (2016, p. 541), namely, *r* = 0.19 (*p* < 0.01), and the result of our replication study. Both fixed-effect and random-effects meta-analytic models were considered. Cross-study effect homogeneity was assessed using the Cochrane *Q* test and the *I*^2^ statistic, with the latter one being an effect-size metric for effect-size heterogeneity (Higgins et al., 2003). These meta-analyses were carried out using the R package meta (Schwarzer, 2007).

To further evaluate the findings regarding the correlation between weekly TV consumption and the dichotomized death penalty questionnaire sum score, we calculated the effect size for which the original study would have had 33% power to detect it (the so-called r33% in the small-telescopes analysis method, as introduced by Simonsohn, 2015), based on the sample size of the Till et al. (2016) study. Then, the result of the replication study, along with its 90% and 95% *CI*s, was compared to this *r*_33%_ statistic (i.e., the smallest effect size possibly detectable by the original study), to check whether the original study, with its smaller sample size (metaphorically, the “small telescope”; see Simonsohn, 2015) had enough power at all to detect an effect of the size observed in the replication study, with its larger sample size (metaphorically, a “larger telescope”). In addition, the *CI* computed for the replication study result served to evaluate whether this interval estimate around the point estimate included zero or not.

#### Assumptions Violated in the Dataset Vis-à-vis the Planned Data Analyses

The weekly number of hours spent watching TV was not normally distributed, showing a positive skew of 2.02 (*SE* = 0.1) and a kurtosis of 6.82 (*SE* = 0.2). In similar vein, a departure from the normal distribution was apparent for the weekly number of hours spent online streaming, with a positive skewness of 2.25 (*SE* = 0.1) and a kurtosis of 8.81 (*SE* = 0.2). Further analysis indicated that TV viewing had *n* = 6 extreme outliers (>3 *SD*s above the mean) and online streaming *n* = 10 such outliers. Hence, model assumptions for the Pearson correlation coefficients appeared not to be sufficiently met with these data, a scenario not accounted for in the preregistered analysis plan. Consequently, all tests of the close replication part were calculated with the untransformed and with the log-transformed study variables TV viewing and online streaming. A comparison of the respective results indicated no substantial differences between these, except that the correlation coefficient between the dichotomized death penalty questionnaire sum score and TV viewing reached nominal significance only with the log-transformed variable. The same applied to the *p*-value for TV viewing in the logistic regression model with the dichotomized death penalty questionnaire sum score as outcome. Apart from these minor differences, we applied the analysis as planned and preregistered; to provide full transparency on the data analysis, all results of the close replication part with log-transformed values are reported in Supplementary Tables S4, S5.

### Exploratory Analysis

The following supplemental analyses were not preregistered, but conducted as well. Pearson correlations were calculated to further investigate the relationship between age and suggestibility, and between the sum score of the 5-item death penalty questionnaire and suggestibility. *P*-values were Bonferroni-adjusted, as suggestibility was tested twice against other variables.

In a second step, the logistic regression models of the conceptual extension part were further extended by including the dichotomous variable nationality (Austrian vs. not) as a further predictor, because *n* = 80 participants reported non-Austrian citizenships.

*Post hoc* power analyses investigated the results of the preregistered meta-analysis further, by calculating the statistical power the original study and the replication attempt would have had to detect an effect of the size indicated by the meta-analytic estimates.

Since the present study revealed age as the strongest predictor for at least one incorrect answer in the 5-item questionnaire, another meta-analysis was carried out, to get an estimate of the association of respondent age with at least one incorrect response in the death penalty questionnaire.

## Results

### Confirmatory (Preregistered) Analyses

On average, respondents spent 12.02 h (*SD* = 12.42) watching TV and 9.36 h (*SD* = 9.82) using online streaming services weekly. The amount of hours spent for TV viewing ranged from 0 to 90 h (*Mdn* = 10), and from 0 to 70 h (*Mdn* = 7) for the usage of online streaming services. Weekly TV consumption was significantly higher than online streaming: paired *t*(596) = 4.42, *p* < 0.001, *d* = 0.18. Table 1 provides the percentages of incorrect and correct responses for each item about death penalty in Austria, alongside average duration of weekly TV viewing and online streaming. The likelihood to respond to the direct question on the death penalty in Austria incorrectly increased (nominally significantly, but practically not relevant) with giving at least one wrong response in the 5-item questionnaire, *OR* = 1.04, *p* < 0.001, 95% *CI* [1.02,1.10].

**TABLE 1:** Death penalty questionnaire performance, weekly TV viewing, and streaming (*N* = 597).

				**TV viewing**		**Streaming**	
**Question**	**Answer**	***N***	**%**	***M***	***SD***	***M***	***SD***
Death row	Correct	581	97.3	11.89	12.42	9.23	9.61
	Incorrect	16	2.7	16.43	11.93	14.38	15.16
Lethal 5 year	Correct	585	98	11.91	12.42	9.35	9.85
	Incorrect	12	2	17.25	12.02	9.92	8.41
Lethal 25 year	Correct	544	91.1	11.86	12.53	9.39	9.9
	Incorrect	53	8.9	13.65	11.19	9.12	9.04
Electric 5 year	Correct	587	98.3	11.91	12.41	9.33	9.84
	Incorrect	10	1.7	18.3	12.42	11.6	8.36
Electric 25 year	Correct	564	94.5	11.71	12.35	9.51	9.94
	Incorrect	33	5.5	17.3	12.67	6.91	7.19
5-item score^a^	Correct	538	90.1	11.73	12.42	9.33	9.66
	Incorrect	59	9.9	14.58	12.03	9.72	11.22
Death penalty^b^	No	595	99.7	12.03	12.43	9.39	9.82
	Yes	2	0.3	7	9.9	0.75	1.06

*Table entries show the prevalence of correct and incorrect answers for each question, along with means (*M*) and standard deviations (*SD*) of weekly TV viewing and online streaming. For the sake of comparability with the original study, as attempted to replicate here, the table layout formatting follows Till et al. (2016, p. 541, their Table 1); cells marked in gray report results from the conceptual extension part of the replication study. ^a^ Overall performance on the questionnaire on death penalty in Austria (α = 0.82), coded as correct, if all five items were answered correctly, and the 95% *CI* for the prevalence of at least one incorrect answer was [7.7, 12.1]. ^*b*^Direct question on current death penalty practices in Austria, with “No” representing the correct answer. The bootstrapped 95% *CI*, based on 1000 samples, for the prevalence of an incorrect answer to the direct question was [0.0, 0.8].*

In terms of their TV viewing and streaming habits, respondents most frequently ranked comedy, drama, and international crime and detective series under their top-three preferred TV genres they watched (see Supplementary Table S1 for details). Approximately 40% of respondents reported to watch international crime and detective series regularly; of these, 7.1% responded incorrectly to at least one of the five items of the death penalty questionnaire. Conversely, 11.6% of respondents who did not list international crime and detective series as their top-three preferred TV genres (*n* = 371) also gave at least one incorrect answer in the 5-item questionnaire. Preference of international crime and detective series was not associated with the performance in the 5-item questionnaire, *OR* = 0.58, *p* = 0.07, 95% *CI* [0.32,1.06].

The intercorrelation matrix of all study variables in the close replication part (Table 2) shows that only respondent age was consistently associated with correct responses on all five items and with the dichotomized sum score of the 5-item death penalty questionnaire. Older respondents were more likely to answer these questions correctly. TV viewing correlated with merely one out of the six dependent death penalty measures. Online streaming did not significantly correlate with respondents’ overall performance in the death penalty questionnaire (*r* = −0.001, *p* = 0.98, 95% *CI* [−0.09,0.08]). Neither TV viewing (*r* = −0.02, *p* = 0.57, 95% *CI* [−0.1,0.06], nor online streaming (*r* = −0.05, *p* = 0.21, 95% *CI* [−0.13,0.03]) correlated with the direct question on death penalty in Austria. However, it is emphasized that the prevalence of incorrect answers on the direct question about death penalty was very low (see Table 1).

**TABLE 2:** Intercorrelations between all measures of the close replication study (*N* = 597).

**Variable**	**1**		**2**	**3**	**4**	**5**	**6**	**7**	**8**
1. TV viewing		.							
2. Age	0.09^*^	[−0.002,0.20]	.						
3. Education	**−0.25^∗∗∗^**	**[−0.34,−0.17]**	−0.13^∗∗^ [−.22,−0.03]	.					
4. Death Row	0.06	[−0.02,0.14]	−0.08^*^ [−0.15,0.01]	**−0.18^∗∗∗^ [−.28,−0.07]**	.				
5. Electric 5 year	0.07	[−0.01,0.15]	−0.10^*^ [−0.16,−0.01]	−0.12^∗∗^ [−0.22,−0.01]	**0.63^∗∗∗^ [0.39,0.80]**	.			
6. Electric 25 year	0.10^*^	[0.02,0.19]	**−0.16^∗∗∗^ [−0.23,−0.08]**	−0.06 [−.15,−0.02]	**0.37^∗∗∗^ [0.18,0.54]**	**0.54^∗∗∗^ [0.37,0.68]**	.		
7. Lethal 5 year	0.06	[−0.01,0.14]	**−0.14^∗∗∗^ [-.18, -.09]**	**−0.17^∗∗∗^ [−0.28,−0.04]**	0.**64^∗∗∗^ [0.41,0.83]**	**0.82^∗∗∗^ [0.59,0.69]**	**0.49^∗∗∗^ [0.30,0.64]**	.	
8. Lethal 25 year	0.04	[−0.03,0.12]	**−0.20^∗∗∗^ [−.26,−0.14]**	−0.03 [−0.11,0.05]	**0.42^∗∗∗^ [0.27,0.55]**	**0.42^∗∗∗^ [0.28,0.54]**	**0.70^∗∗∗^ [0.59,0.80]**	**0.46^∗∗∗^ [0.32,0.58]**	.
9. 5-item score^a^	0.07	[−0.01,0.15]	**−0.19^∗∗∗^ [−0.27,−0.13]**	−0.07 [−0.16,0.02]	**0.50^∗∗^ [0.38,0.60]**	**0.39^∗∗∗^ [0.26,0.51]**	**0.73^∗∗∗^ [0.64,0.82]**	**0.43^∗∗∗^ [0.30,0.55]**	**0.94^∗∗∗^ [0.89,0.98]**

Table entries are Pearson correlation coefficients (*r*) for continuous study variables (age, education, TV viewing), and point-biserial correlation coefficients (*r*_*pb*_) for dichotomous study variables (the death penalty questionnaire items). Bootstrapped 95% *CI*s, based on 1000 samples, are reported in brackets. ^*a*^Overall performance on the questionnaire on death penalty in Austria (α = 0.82), coded as correct, if all five items (study variables 4–8) were answered correctly. ^*^p < 0.05, ^∗∗^*p* < 0.01, ^∗∗∗^*p* < 0.001 (two-tailed). Items remaining nominally significant with Bonferroni-adjusted α of 0.0014 (0.05/36) are in boldface.

The logistic regression models (Table 3; for more details, see Supplementary Table S2) suggested that TV viewing did not predict performance on the death penalty questionnaire, when respondent age and education were accounted for. TV viewing only achieved nominal significance as a predictor for the electric chair (last 25 years) item. In contrast, respondent age was significantly negatively related to performance in the death penalty questionnaire. This was also the case when suggestibility, the duration participants spent living in Austria, and whether they regularly watched international crime and detective series vs. not were accounted for in the model. A logistic regression model with the direct question on death penalty in Austria as a predictor was not significant: χ^2^(3, 597) = 3.57, *p* = 0.31, Nagelkerke *R*^2^ = 0.14.

**TABLE 3:** Results of the binary logistic regression analyses predicting beliefs about Austrian death penalty practices (*N* = 597).

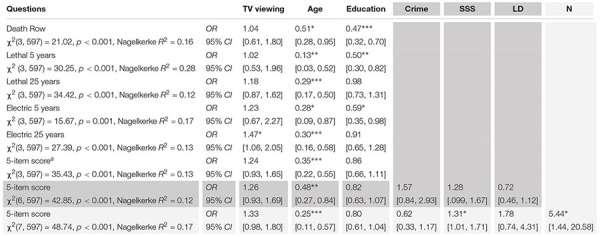

Table entries are odds ratios (*OR*) and 95% confidence intervals (*CI*). ^*a*^Overall performance on the questionnaire on death penalty in Austria (α = 0.82), coded as correct, if all five items were answered correctly. The conceptual and extension part of the replication study (highlighted in dark-gray) introduced whether participants mostly watched international crime series (*Crime*), suggestibility (*SSS*), and living duration in Austria (*LD*) as covariates, the exploratory analysis part (highlighted in light-gray) added Austrian (vs. non-Austrian) nationality (*N*) as covariate. Following the original study (Till et al., 2016), continuous variables were standardized before entered into the models. All logistic regression models remained significant with Bonferroni-adjusted α of 0.01 (0.05/6). ^*^p < 0.05, ^∗∗^*p* < 0.01, ^∗∗∗^*p* < 0.001 (two-tailed).

None of the respondents gave inconsistent responses in the 5-item questionnaire, i.e., answering the questions about the electric chair/lethal injection in the past 5 years incorrectly, but those about the past 25 years correctly (see Supplementary Table S3).

The Mokken scale analysis results did not suggest substantial violations of item transitivity, and all five items appeared to form one meaningful scale, thus indicating that in this sample the 5-item questionnaire apparently measured one underlying latent variable. Pairwise item homogeneity coefficients (*H*_*ij*_) for all item pairs were > 0.30, ranging from 0.54 to 1.00. Individual item homogeneity indices (*H*_*i*_) all were > 0.70, and the homogeneity coefficient for the entire scale was *H* = 0.84 (*SE* = 0.05). Hence, monotonicity was the case, and the 5-item death penalty questionnaire appeared to be a “strong” Mokken scale (see Table 4).

**TABLE 4:** Results of the Mokken scale analysis of the death penalty questionnaire.

		***H*_*ij*_**			
	***H*_*i*_**	**1**	**2**	**3**	**4**
1. Death Row	0.71 (0.09)	.			
2. Lethal 5 years	0.86 (0.07)	0.74 (0.13)	.		
3. Lethal 25 years	0.89 (0.04)	0.79 (0.11)	1.00 (0.00)	.	
4. Electric 5 years	0.92 (0.05)	0.79 (0.13)	0.90 (0.10)	1.00 (0.00)	.
5. Electric 25 years	0.82 (0.05)	0.54 (0.13)	0.82 (0.11)	0.90 (0.06)	1.00 (0.00)

*Table entries are homogeneity coefficients (*H*_*i*_) for each item, as well as for each item pair (*H*_*ij*_), accompanied by their standard errors (*SE*) in parentheses.*

**FIGURE 1:**
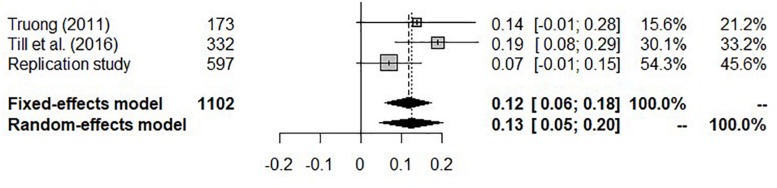
Forest plot for individual study results on the associations (Pearson correlation coefficients, *r*, along with 95% confidence intervals, *CI*) between TV viewing and performance on the death penalty questionnaire, and meta-analytic quantification of the evidence for these associations. Study weight refers to the weight each study is assigned in fixed-effect and random-effects meta-analytic models.

Cumulating the results from Truong (2011), the original study (Till et al., 2016), and the current replication attempt meta-analytically yielded a nominally significant, but small, positive correlation between weekly TV viewing and at least one incorrect response in the 5-item death penalty questionnaire. Overall effect heterogeneity across studies was *I*^2^ = 37.8%, 95% *CI* [0.0,80.04]. A non-significant *Q*(2) = 3.21, *p* = 0.20 suggested that assuming effect homogeneity across studies was tenable. Fixed-effect and random-effects meta-analyses yielded numerically very similar effect-size estimates and associated 95% *CI*s (Figure 1).

**FIGURE 2:**
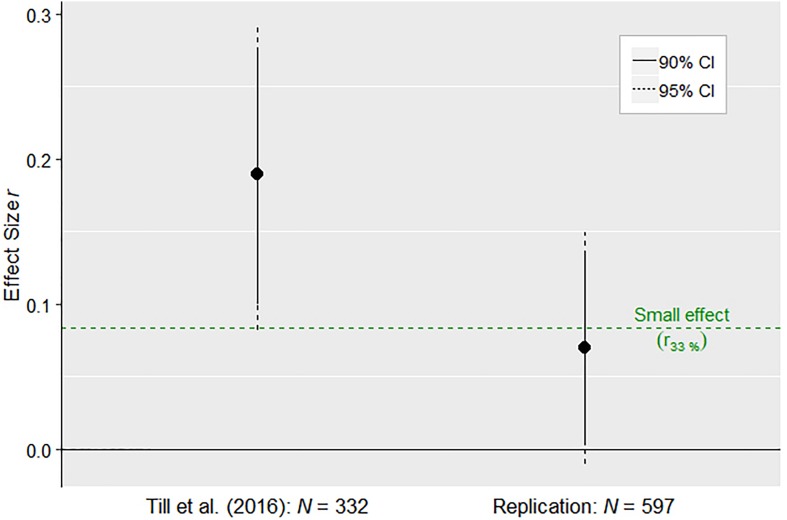
Results of the small-telescopes analysis, evaluating the detectability level of the effect in the original study (Till et al., 2016), according to study design and sample size, in the light of the effect size observed in the replication study. Displayed are the effect sizes (Pearson *r)* observed in the original vs. in the replication study, accompanied by the 95% and 90% confidence intervals (*CI*). The colored horizontal line displays the smallest possibly detectable effect in the original study (*r*_33%_), according to its sample size (operationalized as the original study having 33% statistical power; Simonsohn, 2015).

According to the small-telescopes anlaysis, the effect size for which the original study of Till et al. (2016) would have had 33% power to detect it (i.e., the smallest possibly detectable effect), amounted to *r*_33%_ = 0.08. Comparing the replication result to the original study result suggested that the Till et al. (2016) study would have been sufficiently powered to detect an effect of the size as the one in the replication attempt. However, in contrast to the original study, the 95% *CI* for the effect observed in the replication study included zero and thus nominally was not significant (see Figure 2).

### Exploratory (Not Preregistered) Analyses

Exploratory analysis revealed a significant negative correlation between age and suggestibility (*r* = −0.24, *p* < 0.01, 95% *CI* [−0.31,−0.17]) and a significant positive correlation between suggestibility and the dichotomized sum score of the death penalty questionnaire (*r* = 0.12, *p* < 0.01, 95% *CI*[0.02,0.21]). The Bonferroni-adjusted significance level to account for multiple hypothesis testing was 0.025 (0.05/2).

Participants not holding Austrian citizenship more likely gave at least one incorrect answer in the 5-item questionnaire, even when age, education, whether participants regularly watched crime and detective series or not, and living duration in Austria where accounted for. Furthermore, suggestibility reached significance after adding nationality to the regression equation (Table 3).

**FIGURE 3:**
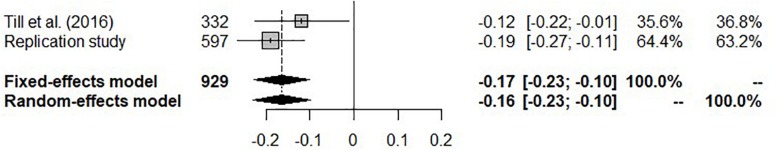
Forest plot for individual study results on the associations (Pearson correlation coefficients, *r*, along with 95% confidence intervals, *CI*) between age and performance on the death penalty questionnaire. The effect describes the relationship between participants’ age and the overall performance in the death penalty questionnaire, and meta-analytic quantification of the evidence for these associations. Study weight refers to the weight each study is assigned in fixed-effect and random-effects meta-analytic models.

The exploratory meta-analysis cumulated the effects from the original and the replication study regarding the association of respondent age and at least one incorrect answer in the death penalty questionnaire. Overall, cross-study effect heterogeneity was *I*^2^ = 8.3%, and the not significant *Q*(1) = 1.09, *p* = 0.30 indicated that effect homogeneity across studies could be assumed. Fixed-effect and random-effects meta-analytic results had nearly the same point estimates and identical 95% confidence intervals (Figure 3). The sample of Truong (2011) was not included in this additional meta-analysis, because it was comprised of undergraduate students and thus uninformative for investigating effects of respondent age.

## Discussion

### Confirmatory (Preregistered) Analysis

In a two-part endorsed, prereviewed, and preregistered replication study, complemented with a preregistered meta-analysis, we tested the hypothesis that the amount of weekly TV viewing would influence mistaken beliefs about Austria still or recently practicing the death penalty (Till et al., 2016). The first part, a close replication, investigated the replicability of the original effect by using exactly the same measures and methods as the original study. The conceptual extension part served to test the generalizability of the original hypothesis, to account for possible confounds, and to investigate the possibility that the effect, due to faulty design of the psychometric measure (5-item death penalty questionnaire) used in the original study, could be spurious. Finally, the effect observed in the close replication part, vis-à-vis the properties and findings of the original study, was evaluated by conducting a small-telescopes analysis and a meta-analysis of all three existing datasets (Truong, 2011; Till et al., 2016; and the current replication attempt). All in all, the results thereof are mixed, providing partial support for the original hypothesis, but as well for probable spuriousness of the effect.

#### Close Replication Part

In terms of the close replication results, the present study replicated the prevalence of at least one incorrect answer in the 5-item death penalty questionnaire. However, contrary to the original study, weekly duration of TV viewing was not a salient predictor of the questionnaire’s outcome. Instead, respondent age predicted the probability of at least one incorrect answer, even when accounting for TV viewing and education. These results strongly indicate that older individuals more likely answered the questionnaire correctly. A similar age effect was observed by Till et al. (2016), but TV viewing appeared to be the stronger predictor there. In the present study, TV viewing significantly correlated with one of the six dependent variables (or two out of six after applying log-transformation, see Supplementary Tables S4, S5). In addition, the 95% *CIs* of the effects of the replication and of the original study overlapped and the small-telescopes analysis suggested that the original study would have had enough power to detect an effect of the size of the one obtained in the replication study.

Participants in the replication attempt reported watching more television than participants in the original study (merely *M* = 5.11, *SD* = 3.52 h per week; Till et al., 2016, p. 541) and were more representative of the overall population, as the general Austrian population is estimated to spend approximately 3 h a day watching TV (Oesterreichischer Rundfunk [ORF], 2016). However, the positive skew for the TV watching variable also indicated that a number of participants spent little to no time watching TV. Thus, the replication study should have been able to detect effects of TV consumption more clearly, due to the greater sample heterogeneity, but instead correlations were smaller and mostly not significant. Age, on the other hand, gained significance, as compared with the original study, and the correlation coefficient was larger.

Interpreting each item of the 5-item questionnaire individually seemed less informative, due to the strong item-intercorrelations and the high internal consistency of this scale, both in the original and the present study. Another aspect that should be taken into account is the fact that testing one variable against multiple correlated dependent outcomes may dramatically increase the false-positive rate among any findings (Simmons et al., 2011). In light of the scale’s high internal consistency, strong item-intercorrelations, and its strong conformity with the Mokken scale model, one may not rule out that the few significant correlations of TV viewing with individual items may have reached significance merely by chance. A third argument against interpreting each item separately is the low prevalence of incorrect answers and therefore the small observed variance in responses on some of the items (Table 1).

Revisiting the evaluation using the small-telescopes analysis (Simonsohn, 2015), the original study appeared to have had enough power to detect the close replication result, or at least the upper range of the 95% *CI* associated with this effect. However, as mentioned above, the 95% *CI* for the replication study effect not only included *r*_33%_, but also zero (see Figure 2). Hence, the size of this effect is not clearly distinct from zero. Log-transforming the TV viewing variable led to a 95% *CI* that did not include 0 (Supplementary Table S5). However, regardless of the log-transformation, in both data-analytic scenarios the confidence interval from the replication study did not include the point estimate of the effect size from the original study point. In the preregistered meta-analysis, both random-effects and fixed-effect analyses resulted in the same point estimate which significantly differed from zero. However, a post-hoc power analysis showed that the probability of the original study to detect an effect of this size was more or less similar to a coin toss. In contrast to the replication attempt, the meta-analysis supported the original study’s hypothesis that there is a relationship between the weekly amount of TV viewing and erroneous beliefs about existing death penalty in a country which, in actual fact, has abolished it decades ago. Nonetheless, this effect may well be due to a faulty construction of the death penalty questionnaire, a supposition that the conceptual extension part aimed to scrutinize in more detail.

#### Conceptual Extension Part

Till et al. (2016) argued that the correlation between TV viewing and at least one incorrect answer in 5-item death penalty questionnaire is due to the fact that study participants (or Austrians in general) primarily would watch American crime and detective series and might therefore confuse what they see on TV with what is real. The conceptual extension part revealed that American crime and detective series was the third-most popular TV genre. However, whether or not participants regularly watched American crime and detective series did not influence their performance in the death penalty questionnaire. This finding supports similar evidence from Truong (2011), who did not find a significant effect for the amount of American law shows watched.

Truong (2011) and Till et al. (2016) based their assumptions on a theory that was developed in the late 1970s (Gerbner, 1977), where the only medium to watch TV was the classic analog TV at home, or to go to the cinema. Nowadays, the concept of watching TV is much more complex, with services like streaming websites (see Morgan et al., 2015, for a discussion of cultivation theory and new media). Hence, assumptions about the influence of supposedly primarily watched TV cannot be simply drawn from TV programs.

Regarding the generalizability of the original effect, online streaming did not correlate with the results of the death penalty questionnaire. According to Morgan et al. (2015, p. 687) “watching broadcast or cable programs on the Internet is still fundamentally watching TV.” Even though participants spent fewer hours using streaming services than watching TV per week, this does not explain why with the streaming variable the original effect was not preserved. The question on TV viewing belonged to the close replication part of the survey, and the question on streaming to the conceptual extension part of the survey. Consequently, participants had to answer the TV viewing questions before they saw or knew about the streaming item. They also were not allowed to go back afterward. Thus, it might be possible that some of the participants already included the amount they spent online streaming in answering the TV viewing question. Nevertheless, the only consequence would be that TV viewing and streaming were not sufficiently differentiated in both the original and close replication studies. However, the fact that both variables differed in their associations with the results of the death penalty questionnaire in the present study counteracts this argument. Furthermore, both TV viewing and streaming did not correlate with the result of the direct question on the death penalty in Austria.

#### Response Pattern on the Death Penalty Questionnaire

Mokken scale analysis demonstrated that the five items of the death penalty questionnaire formed a common scale and collectively measured one underlying latent variable. However, the question remains whether this underlying variable really are mistaken beliefs, or rather consistent response behaviors in answering the questions on recent and current practices of capital punishment in Austria alike (either correctly or incorrectly). Considering that only a very small fraction of respondents answered the direct question about death penalty in Austria incorrectly, it is doubtful that the questionnaire should have measured the same underlying variable.

Furthermore, the prevalence of incorrect answers was higher for the questions about the last 25 years compared to the last 5 years. Leading to the idea that respondents could have been insecure, due to the suggestive and repetitive nature of the questionnaire, rather than actually thinking that Austria recently practiced the death penalty. As described in Tourangeau and Rasinski (1988), prior items in a questionnaire can influence the answers given to following ones. Based on the answered items the respondent forms a framework to interpret the scope of consecutive questions. In the present study, respondents potentially interpret the purpose of subsequent questions to gather new rather than redundant information. Hence, the observed incorrect answers potentially display the respondents avoidance of redundancy (backfire effect).

Respondent age appeared to be the strongest predictor for the outcome in the death penalty questionnaire. A possible explanation for this finding could be that asking about a timespan even reaching back before one’s own birth, combined with the strongly repetitive nature of the questions, might have induced insecurity in younger respondents’ judgment. Furthermore, respondents intuitively (but incorrectly) could have assumed that at least one of the statements must be true. This kind of a framing effect might have received an additional boost, as each question was accompanied with the example of writing down the number 6, which, in itself, probably and inadvertently so, may well have introduced an undesired anchoring effect (Jacowitz and Kahneman, 1995; see also Klein et al., 2014). In contrast, older respondents more likely answered correctly, as they were in a position to recall the past 25 years. The lack of any anchoring effects could also serve as an explanation for the extremely low prevalence of incorrect answers on the direct question about the current practice of the death penalty in Austria.

### Exploratory (Not Preregistered) Analysis

Non-Austrian respondents more likely gave at least one incorrect answer to the death penalty, and suggestibility gained significance in a regression model that also included nationality. This indicates that the suggestive effects of the specific item wording may have boosted incorrect answers in the questionnaire.

The meta-analytic point estimate for the associations of respondent age and at least one incorrect answer to the questionnaire differed significantly from zero and was stronger than the effect of TV viewing as a predictor for the outcome on the death penalty questionnaire. However, the 95% CIs of these effect sizes overlapped. Interestingly, and in contrast to Till et al. (2016) and the current replication attempt, Truong (2011) found a remarkable high prevalence of false beliefs about current or recent practices of the death penalty in Canada. In the honors thesis 78% out of the 173 participating undergraduate students gave at least one incorrect answer in the death penalty questionnaire, whereas, as mentioned above, TV viewing was not a predictor for this misperception. Hence, TV viewing effects were not robust enough to account for age and education effects within a relatively homogenous student sample. In turn, these observations can also be interpreted in favor of respondent age being a strong predictor of performance in the death penalty questionnaire. Guided by the observed response patterns in our data, we argue that with increasing age especially questions about the last 25 years might become less suggestive, because participants may have an actual recollection of the inquired time span.

### Limitations

The current meta-analyses were based on zero-order correlation coefficients, i.e., without controls for third variables. Hence, the comparability between these meta-analytic estimates may be limited due to possible confounding effects. The proposed interplay between respondent age and trait suggestibility rests on exploratory analyses, which we did not preregister prior to data collection, but only developed after having conducted our planned, preregistered analyses (Kerr, 1998). Nevertheless, respondent age evidently strongly predicted outcomes of the death penalty questionnaire.

One shortcoming was the replication sample’s educational level and typical age. Participants were better educated than in the original study and did not accurately represent the Austrian general population. Educational level may be another predictor for incorrect answers in a more representative sample (Till et al., 2016), and lower education may even increase the prevalence of such incorrect answers. As in Till et al. (2016) the replication sample was rather young, due to the online assessment approach. Thus, the prevalence of incorrect answers might decrease in older-aged samples.

Till et al. (2016) described the 5-item death penalty questionnaire as measuring perceptions of the Austrian justice system. The non-practice of death penalty in Austria since the 1950s undeniably is a distinct difference to America; however, this is only one detail of the Austrian (and American) justice systems. Another aspect is that the sum score for this questionnaire could also be calculated as an actual sum score, i.e., ranging from 0 to 5, to prevent further information loss due to double transformation (i.e., individual item responses dichotomized, from which a dichotomized total score is created). Future research may well profit from constructing a more comprehensive questionnaire, asking about differences between justice systems or even other aspects depicted in TV, such as health care. Furthermore, a more comprehensive assessment of TV viewing that considers actually watched genres and usage of TV in general (entertainment vs. news) would also be beneficial.

Finally, all three studies, so far conducted in this line of research, build on correlational evidence, which does not necessarily imply causality. Considering the smallness of the observed effects, both the feasibility and the cost-benefit ratio of experimental approaches to this topic may well be considered as disputable.

## Conclusion

This replication attempt of the evidence of Till et al. (2016) suggests that the 5-item death penalty questionnaire, as previously used, might have induced insecurity in respondents, thereby triggering incorrect answers. Confronted with a direct question on capital punishment in Austria, nearly no participant provided a wrong answer. TV viewing did not predict incorrect answers in the replication study, whereas preliminary meta-analytic evidence is suggestive for such effects, albeit of trivial size. In conclusion, evidence for a high prevalence of erroneous beliefs about existing capital punishment in a country where it is not practiced since decades may well be a research artifact, due to a faultily designed questionnaire. Any effects of television viewing habits on such erroneous beliefs likely are small at best.

## Ethics Statement

Data collection in this study was fully conducted online. The first page of the survey contained a short study description and information for study participants. The participants provided their consent to partake in the survey via clicking a button, which was also required to start the survey. Participation was anonymous from the outset in this online study; there was no written informed consent. Participation was voluntary and without any numeration. We would like to note that the study participation affected neither the physical or the psychological integrity or the right for privacy, nor the other personal rights or interests of the participants. Such being the case, this study was thus exempt from formal ethical approval according to national laws (Austrian Universities Act 2002).

## Author Contributions

MV conceived the original research idea. MB and MV designed the experiment and authored the replication plan. MB carried out the data collection and data analysis. MV and UT supervised the analysis. MB took the lead in writing the manuscript. MV provided important intellectual content in revising the manuscript. All authors provided critical feedback to shape the final manuscript.

## Conflict of Interest Statement

The authors declare that the research was conducted in the absence of any commercial or financial relationships that could be construed as a potential conflict of interest.
