# Supplementary material for: Does Really One in Ten Believe Capital Punishment Exists in a Contemporary European Community Country? An Endorsed, Prereviewed, Preregistered Replication Study and Meta-Analysis
Source: Front Psychol. 2019 Jul 19;10:1601. doi: 10.3389/fpsyg.2019.01601 (PMC6658835; doi:10.3389/fpsyg.2019.01601)
Supplement: Supplementary file 1 [file Data_Sheet_1.docx]

**Does really one in ten believe capital punishment exists in a contemporary European Community country? An endorsed, prereviewed, preregistered replication study and meta-analysis**

Magdalena Boch*, Ulrich S. Tran and Martin Voracek

Department of Basic Psychological Research and Research Methods, Faculty of Psychology, University of Vienna, Vienna, Austria

Number of tables: 5

***CORRESPONDENCE**

Magdalena Boch, MSc
E-mail: magdalena.boch@univie.ac.at

## S1 Supplementary tables

| Table S1 |  |  |
| --- | --- | --- |
| *Television (TV) and film genre ranking* | | |
| Genre | N | % |
| Comedy | 350 | 19.54 |
| Drama | 267 | 14.91 |
| Crime and detective international | 226 | 12.62 |
| Science fiction / Fantasy | 165 | 9.21 |
| Crime and detective national / European | 151 | 8.43 |
| Historical | 144 | 8.04 |
| Action | 125 | 6.98 |
| Mystery / Horror | 95 | 5.30 |
| Hospital / Doctor | 92 | 5.14 |
| Soap / Telenovela | 85 | 4.75 |
| Animation | 58 | 3.24 |
| “Heimat” / Family | 33 | 1.84 |
| *Note*. Table entries are absolute numbers (*N)* and percentages (%) of the top three primarily watched TV genres, as selected as participants, and ranked in descending order. Each participant (*N* = 597) chose three genres; hence, total *N* = 1791. | | |

| Table S2 | | | | |
| --- | --- | --- | --- | --- |
| *Detailed version of Table 3, including the standardized regression coefficients (β), along with their standard errors* (*SE*), *for the close replication measures.* | | | | |
| Questions |  | TV viewing | Age | Education |
| Death Row: $\chi^{2}$(3, 597) = 21.02, *p* < .001, Nagelkerke *R^2^* = .16 | *OR* | 1.04 [0.61, 1.80] | 0.51 [0.28, 0.95] | 0.47 [0.32, 0.70] |
|  | β | 0.04 | -0.67* | -0.76*** |
|  | *SE* | 0.28 | 0.32 | 0.20 |
| Lethal 5 yr.: $\chi^{2}$(3, 597) = 30.25 , *p* < .001, Nagelkerke *R^2^* = .28 | *OR* | 1.02 [0.53, 1.96] | 0.13 [0.03, 0.52] | 0.50 [0.30, 0.82] |
|  | β | 0.02 | -2.07** | -0.70** |
|  | *SE* | 0.33 | 0.72 | 0.26 |
| Lethal 25 yr.: $\chi^{2}$(3, 597) = 34.42 , *p* < .001, Nagelkerke *R^2^* = .12 | *OR* | 1.18 [0.87, 1.62] | 0.29 [0.17, 0.50] | 0.98 [0.73, 1.31] |
|  | β | 0.17 | -1.24*** | -0.02 |
|  | *SE* | 0.16 | 0.27 | 0.15 |
| Electric 5 yr.: $\chi^{2}$(3, 597) = 15.67 , *p* = .001, Nagelkerke *R^2^* = .17 | *OR* | 1.23 [0.67, 2.27] | 0.28 [0.09, 0.87] | 0.59 [0.35, 0.98] |
|  | β | 0.21 | -1.29* | -0.53* |
|  | *SE* | 0.31 | 0.59 | 0.26 |
| Electric 25 yr.: $\chi^{2}$(3, 597) = 27.39, *p* < .001, Nagelkerke *R^2^* = .13 | *OR* | 1.47 [1.06, 2.05] | 0.30 [0.16, 0.58] | 0.91 [0.65, 1.28] |
|  | β | 0.39* | -1.20*** | -0.09 |
|  | *SE* | 0.17 | 0.33 | 0.17 |
| 5-item score^a^: $\chi^{2}$(3, 597) = 35.43 , *p* < .001, Nagelkerke *R*^2^ = .12 | *OR* | 1.24 [0.93, 1.65] | 0.35 [0.22, 0.55] | 0.86 [0.66, 1.11] |
|  | β | 0.21 | -1.05*** | -0.15 |
|  | *SE* | 0.15 | 0.23 | 0.13 |
| *Note*. Table enteries are odds ratios (*OR*) with 95% confidence intervals (*CI*). ^a^ Overall performance on the questionnaire on death penalty in Austria (α = .82), coded as correct, if all five items were answered correctly. Following the original study (Till et al., 2016), continuous variables (age, TV viewing) were standardized before entered into the model.  **p* < .05, ***p* < .01, ****p* < .001 (two-tailed). All logistic regression model results remained nominally significant with Bonferroni-adjusted α level of .01 (.05/5). | | | | |

45

| Table S3 | | | | |
| --- | --- | --- | --- | --- |
| *Crosstabulation of item-response patterns on the lethal injection and electric chair item pairs.* | | | | |
|  |  | Lethal 25 yr. | |  |
|  |  | correct | incorrect | Total *N* |
| Lethal 5 yr. | correct | 544 | 41 | 585 |
|  | incorrect | 0 | 12 | 12 |
| Total *N* |  | 544 | 53 | 597 |
|  |  | Electric 25 yr. | |  |
|  |  | correct | incorrect | Total |
| Electric 5 yr. | correct | 564 | 23 | 585 |
|  | incorrect | 0 | 10 | 10 |
| Total |  | 544 | 33 | 597 |
| *Note.* Table entries are the absolute numbers of correct and incorrect responses on the *lethal injection* and the *electric chair* item pairs. Both item pairs queried the number of inmates sentenced with the respective capital punishment method in the past 5 years and the past 25 years. | | | | |

## S2 Log-transformed analysis of the close replication

| Table S4 | | | | | | | | | |
| --- | --- | --- | --- | --- | --- | --- | --- | --- | --- |
| *Intercorrelation matrix of study variables with log-transformed TV viewing (N = 597).* | | | | | | | | | |
| Variable | 1 | | 2 | 3 | 4 | 5 | 6 | 7 | 8 |
| 1. TV viewing^a^ | . | |  |  |  |  |  |  |  |
| 2. Age | .08* | [0.00, .16] | . |  |  |  |  |  |  |
| 3. Education | **-.23***** | **[-.30, -16]** | -.13** [-.22, -.03] | . |  |  |  |  |  |
| 4. Death Row | .04 | [-.06, .13] | -.08* [-.15; .01] | **-.18*** [-.28, -.07]** | . |  |  |  |  |
| 5. Electric 5 yr. | .05 | [-.04, .13] | -.10* [-.16, -.01] | -.12** [-.22, -.01] | **.63*** [.39, .80]** | . |  |  |  |
| 6. Electric 25 yr. | .11** | [.04, .18] | **-.16*** [-.23, -.08]** | -.06 [-.15, -.02] | **.37*** [.18, .54]** | **.54*** [.37, .68]** | . |  |  |
| 7. Lethal 5 yr. | .06 | [-.04, .13] | **-.14*** [-.18, -.09]** | **-.17*** [-.28, -.04]** | .**64*** [.41, .83]** | **.82*** [.59, .69]** | **.49*** [.30, .64]** | . |  |
| 8. Lethal 25 yr. | .07 | [-.001, .14] | **-.20*** [-.26, -.14]** | -.03 [-.11, .05] | **.42*** [.27, .55]** | **.42*** [.28, .54]** | **.70*** [.59, .80]** | **.46*** [.32, .58]** | . |
| 9. 5-item score^b^ | .08* | [.01, .16] | **-.19*** [-.27, -.13]** | -.07 [-.16, .02] | **.50** [.38, .60]** | **.39*** [.26, .51]** | **.73*** [.64, .82]** | **.43*** [.30, .55]** | **.94*** [.89, .98]** |
| *Note.* Table entries are Pearson correlation coefficients (*r*) for continuous study variables (age, education, TV viewing) and point-biserial correlation coefficients (*r*_pb_) for dichotomous study variables (the death penalty questionnaire items). Bootstrapped 95% *Ci*s for the correlation coefficients, based on 1000 samples, are reported in brackets. ^a^Log-transformed variable, to account for distributional skewness. ^b^Overall performance on the questionnaire on death penalty in Austria (α = .82), coded as correct, if all five items (tabulated variables 4. to 8.) were answered correctly.  **p* < .05, * *p* < .01, ****p* < .001 (two-tailed).  Associations remaining nominally significant with Bonferroni-adjusted α level of .0014 (.05/36) are in boldface. | | | | | | | | | |

| Table S5 | | | | |
| --- | --- | --- | --- | --- |
| *Detailed logistic regression results with log-transformed TV viewing (N = 597).* | | | | |
| Questions |  | TV viewing^a^ | Age | Education |
| Death Row: $\chi^{2}$(3, 597) = 21.00, *p* < .001, Nagelkerke *R^2^* = .16 | *OR* | 0.999 [0.64, 1.57] | 0.51 [0.27, 0.95] | 0.46 [0.31, 0.69] |
|  | β | -.001 | -0.67* | -0.77*** |
|  | *SE* | 0.23 | 0.32 | 0.20 |
| Lethal 5 yr.: $\chi^{2}$(3, 597) = 30.40 , *p* < .001, Nagelkerke *R^2^* = .28 | *OR* | 1.12 [0.64, 1.96] | 0.13 [0.03, 0.52] | 0.51 [0.31, 0.84] |
|  | β | 0.11 | -2.08** | -0.67** |
|  | *SE* | 0.29 | 0.73 | 0.25 |
| Lethal 25 yr.: $\chi^{2}$(3, 597) = 36.90 , *p* < .001, Nagelkerke *R^2^* = .13 | *OR* | 1.28 [0.98, 1.66] | 0.29 [0.17, 0.49] | 1.002 [0.75, 1.33] |
|  | β | 0.24 | -1.25*** | 0.002 |
|  | *SE* | 0.13 | 0.27 | 0.15 |
| Electric 5 yr.: $\chi^{2}$(3, 597) = 15.72 , *p* = .001, Nagelkerke *R^2^* = .17 | *OR* | 1.24 [0.67, 2.29] | 0.27 [0.09, 0.86] | 0.59 [0.36, 0.98] |
|  | β | 0.21 | -1.30* | -0.53* |
|  | *SE* | 0.31 | 0.59 | 0.26 |
| Electric 25 yr.: $\chi^{2}$(3, 597) = 31.29, *p* < .001, Nagelkerke *R^2^* = .15 | *OR* | 1.67 [1.14, 2.43] | 0.29 [0.15, 0.57] | 0.93 [0.67, 1.30] |
|  | β | 0.51* | -1.22*** | -0.07 |
|  | *SE* | 0.19 | 0.33 | 0.17 |
| 5-item score^b^: $\chi^{2}$(3, 597) = 37.59 , *p* < .001, Nagelkerke *R*^2^ = .13 | *OR* | 1.29 [1.003, 1.67] | 0.35 [0.22, 0.55] | 0.87 [0.67, 1.12] |
|  | β | 0.26* | -1.06*** | -0.14 |
|  | *SE* | 0.13 | 0.23 | 0.70 |
| *Note*. Table entries are odds ratios (*OR*) and 95% confidence intervals (*CI*). Following the original study (Till et al., 2016), age was standardized before entered into the model. ^a^Log-transformed values, to account for distributional skewness. ^b^Overall performance on the questionnaire on death penalty in Austria (α = .82), coded as correct, if all five items were answered correctly. All logistic regression models remained significant with Bonferroni-adjusted α level of .01 (.05/5).  **p* < .05, ***p* < .01, ****p* < .001 (two-tailed). | | | | |
